# Epigallocatechin-3-Gallate, the green tea polyphenol suppresses expression of CD31 and VEGFR2 and enhances the chemotherapeutic potential of irinotecan in AOM/DSS model of colorectal cancer in mice

**DOI:** 10.1186/s43046-026-00412-4

**Published:** 2026-09-17

**Authors:** Sabana Sargam Rahman, Sukanya Baruah, Nabila Akhtara, Jaydeep Kumar Nath, Manuj Kumar Bharali

**Affiliations:** 1 Dept of Zoology, Gauhati University, Guwahati, India; 2 Dept of Zoology, Salbari College, Salbari, India

**Keywords:** AOM/DSS, Colorectal cancer, Irinotecan, Epigallocatechin-3-gallate, VEGFR2 and CD31

## Abstract

**Background:**

Conventional chemotherapeutic treatment of colorectal cancer causes adverse effects such as severe diarrhoea, mucositis and hepatotoxicity. This study aimed to investigate the anti-carcinogenic potential of epigallocatechin-3-gallate and its effects on major angiogenic markers, alone and in combination with irinotecan in colorectal cancer model.

**Methods:**

Colorectal cancer was induced in mice by injecting azoxymethane and oral administration of dextran sulfate sodium in drinking water as described previously. After 16th week of experimentation, animals were sacrificed, morphometrical assessment was conducted and tissues were processed for histopathological, immunohistochemical and gene expression analysis by qRT-PCR.

**Results:**

Results showed reduction in tumour count, alleviation of colorectal cancer symptoms, improvement in histopathological alterations and goblet cell count with combination therapy. Suppression of key oncogenic and angiogenic markers and reduction in liver injury with combination treatment.

**Conclusion:**

Combined treatment of epigallocatechin-3-gallate with irinotecan enhanced the anti-tumour potential of irinotecan, and inhibited cancer progression and expression of tumour-associated angiogenesis markers. Moreover, treatment associated liver injury was also reduced by epigallocatechin-3-gallate supplementation.

**Supplementary Information:**

The online version contains supplementary material available at https://doi.org/10.1186/s43046-026-00412-4.

## Introduction

Cancer is becoming the leading cause of global morbidity and mortality, with nearly 10 million annual deaths around the world [1]. However, colorectal cancer (CRC) ranks 3rd as a leading cause of death associated with cancer, and its prevalence is rising in both developed and developing countries [2]. Currently, CRC management involves multimodal strategies that incorporate surgery, radiotherapy, cytotoxic chemotherapy, immunotherapy, etc. Despite recent advances in cancer therapies, the treatment outcomes are not satisfactory due to various treatment-related toxicities. Though conventional chemotherapeutics like 5-fluorouracil, leucovorin, oxaliplatin, and irinotecan, etc. markedly improved the survival percentage, however treatment failure and disease recurrence remain a major concern to date [3, 4].

Irinotecan (IRN), a topoisomerase-I inhibitor, is widely used as a first- and second-line chemotherapeutic agent in the management of CRC; exerts its effect by stabilizing the top-I–DNA cleavage complex and inducing lethal DNA strand breaks [5, 6]. Despite its potential chemotherapeutic efficacy, the application of irinotecan is limited by many adverse effects, including diarrhoea, neutropenia, mucositis and hepatotoxicity, which reduce dose intensity, impair patient compliance and contribute to therapeutic resistance [7]. In response to these therapeutic limitations, considerable attention has shifted towards natural phytochemicals and bioactive compounds in cancer therapy as well as in various other afflictions owing to their pleiotropic biological activities, including antioxidant, anti-inflammatory, anti-proliferative, immunomodulatory, pro-apoptotic, anti-angiogenic and anti-metastatic effects, making them promising adjuvants to conventional chemotherapy [8–12].

Among these compounds, natural phytochemicals and bio-active molecules such as curcumin, resveratrol, epigallocatechin-3-gallate (EGCG), etc., have demonstrated their potential to modulate the tumourigenic pathways, thereby amplifying the chemosensitivity while mitigating the drug-mediated toxicities [13]. Many such previous studies have also depicted the diverse pharmacological properties of natural bioactive compounds in various other ailments [14–16].

Among all others, EGCG, the primary catechin of green tea (*Camellia sinensis*), has emerged as a potential anti-neoplastic agent having antioxidant, anti-proliferative, anti-angiogenic, and pro-apoptotic activities [17, 18]. Green tea catechins were also reported to enhance the efficacy of the conventional chemotherapy drug IRN by effectively reducing the tumour burden in the colon, while alleviating the side effects of IRN [19]. Moreover, inhibition of tumour angiogenesis by blocking VEGF (Vascular endothelial growth factor) initiated signalling pathway is a major focus of targeted cancer therapy [20]. Tumours like CRC are highly dependent on VEGF and its receptor induced angiogenesis and hence might have a relatively satisfactory response against anti-VEGF drugs [21].

Previous study reported combination of targeted therapy with conventional chemotherapy significantly enhances overall survival compared to chemotherapy alone in CRC patients [22]. Notably, till date, many in vitro studies have investigated the anti-angiogenic potential of EGCG in various types of cancers such as breast, lung and hepatocellular cancer [23]. This provided a strong rationale for combining EGCG with conventional chemotherapeutic drugs, to enhance their therapeutic efficacy in management of CRC. The present study aims to investigate the anti-carcinogenic effects of combination therapy of EGCG with IRN in the AOM/DSS (Azoxymethane/ Dextran sulfate sodium) -induced CRC model with a focus on its effect on major angiogenic markers.

## Materials and methods

### Chemicals and reagents

Azoxymethane (AOM; CAS 25843-45-2), Epigallocatechin-3-gallate (EGCG; CAS 989-51-5) was obtained from Sigma-Aldrich (USA), Dextran sulfate sodium (DSS; CAS 9011-18-1) was purchased from Sisco Research Laboratories (SRL) Pvt. Ltd and Irinotecan (IRN) was sourced from Zydus Oncosciences. All the primary antibodies and kits for immunohistochemistry (IHC) were purchased from Proteintech, USA (Table 1). The kits for estimation of serum alanine transaminase (ALT, Cat. No. 1102210075), aspartate aminotransferase (AST, Cat. No. 1102200075), alkaline phosphatase (ALP, Cat. No. B062404) and total bilirubin (TBIL, Cat. No. 1101050035) were procured from coral clinical systems, India. Rest of the chemicals and reagents were of analytical grade purity and obtained from local commercial suppliers.

**Table 1:** Lists of antibodies and kits for IHC

Primary antibody	Brand name	Dilution	Antibody validation
IHCeasy PCNA Ready-to-use IHC Kit (Cat no. KHC2182)	Proteintech, IL, USA	Ready-to-use	IHCeasy PCNA Ready-to-use IHC Kit KHC2182 | Proteintech https://www.ptglab.com/products/IHCeasy-PCNA-Ready-To-Use-IHC-Kit-KHC2182.htm
Cleaved Caspase 3 Polyclonal antibody (Cat no. 25128-1-AP)	Proteintech, IL, USA	1:250	Cleaved Caspase 3 Polyclonal antibody (25128-1-AP) | Proteintech https://www.ptglab.com/products/cleaved-Caspase-3-Antibody-25128-1-AP.htm
CD31 Polyclonal antibody (Cat no. 28083-1-AP)	Proteintech, IL, USA	1:4000	CD31 Polyclonal antibody (28083-1-AP)) | Proteintech https://www.ptglab.com/products/Cd31-Antibody-28083-1-AP.htm
VEGFR2/ Kinase insert domain receptor Polyclonal antibody (Cat no. 26415-1-AP)	Proteintech, IL, USA	1:500	VEGFR2/KDR Polyclonal antibody (26415-1-AP) | Proteintech https://www.ptglab.com/products/VEGFR2-Antibody-26415-1-AP.htm
IHC Prep & Detect Kit for Rabbit Primary Antibody (Cat no. PK10017)	Proteintech, IL, USA	-	IHC Prep & Detect Kit for Rabbit Primary Antibody PK10017 | Proteintech https://www.ptglab.com/products/IHC-Prep-Detect-Kit-for-Rabbit-Primary-Antibody-PK10017.htm

### Experimental animals and design

**Fig. 1:**
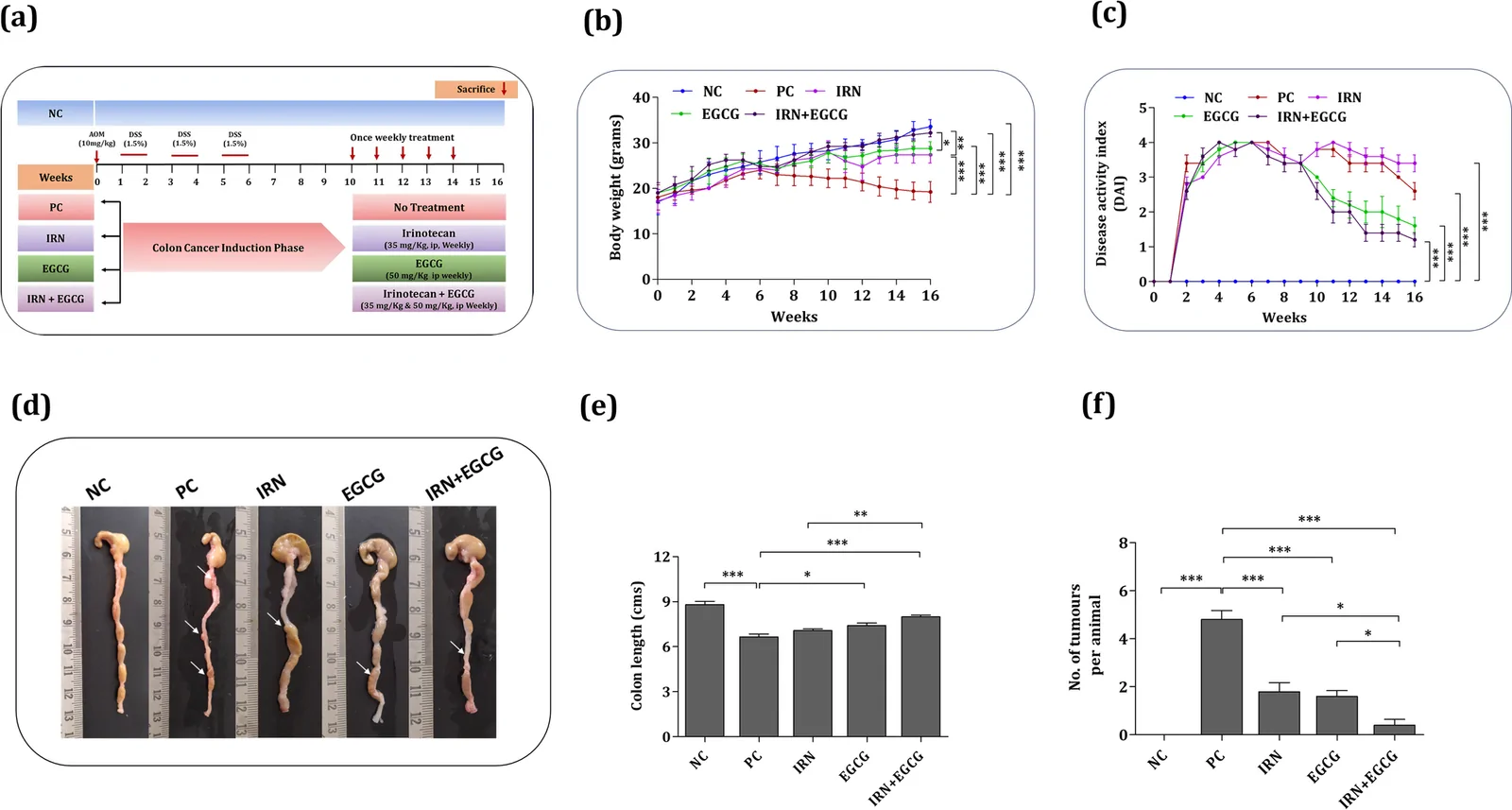
(**a**) Experimental design of the 16-week study. Morphometric observations including (**b**) Changes in body weight and (**c**) DAI score in different treated groups during the experimental period. **d** Gross macroscopical examination of colon from different groups. **e** changes in the colon lengths and (**f**) tumour burden in different treated groups. *** *p* < 0.001, ** *p* < 0.01, * *p* < 0.05

Male Swiss albino mice (*Mus musculus*, *n* = 25, 20 ± 5 g, 10–12 weeks old) were used for this experiment and obtained from the stock Animal House Facility, Department of Zoology, Gauhati University. The experiment was performed following the guidelines of the Institutional Animal Ethical Committee (IAEC), Gauhati University (IAEC/ Per/2024/PP-IAEC/ 2024-8-8/8). All the animals were kept in poly-propylene cages and maintained under controlled laboratory conditions of optimum temperature (24 ± 2 °C) and humidity (56–65 mmHg) with free access to food and water ad libitum. Inclusion criteria comprised of healthy, pathogen-free male mice within the specified age and weight range, while exclusion criteria included animals exhibiting any pre-treatment illness and all animals were monitored daily using a clinical scoring system with predefined humane endpoints, none of which were met during the study (> 20% weight loss, moribund state/distress score > 6). After an acclimatisation period of one week, all the mice were randomly divided into five groups using random numbers generated by the RAND() function in Microsoft Excel; *n* = 5/group, NC (Normal control), PC (Positive control), IRN (Irinotecan), EGCG (Epigallocatechin-3-gallate) and IRN+EGCG and the experiment was performed as per Fig. 1a. The sample size (*n* = 5) was determined based on prior similar in vivo studies and standard practices in preclinical research, which indicate that this number is sufficient to detect biologically relevant differences while minimizing animal use in accordance with animal ethical guidelines of IAEC. Except for the NC group, CRC was induced in all other groups of animals by injecting a single intraperitoneal (i.p.) dose of azoxymethane (AOM; 10 mg/kg) on day 0, followed by three weekly alternative cycles of 1.5% dextran sulphate sodium salt (DSS) in drinking water as reported previously with slight modification [24]. After 10th week, the NC and PC groups received no treatment, the IRN group received 35 mg/kg of irinotecan (i.p.); EGCG group received 50 mg/kg of EGCG (i.p.), and mice in the IRN+EGCG group received irinotecan (35 mg/kg, i.p.) and EGCG (50 mg/kg, i.p.) as separate intraperitoneal injections and were administered simultaneously once weekly from weeks 10 to 14. The selected dosages of IRN (35 mg/kg) and EGCG (50 mg/kg) were derived from prior published studies indicating their therapeutic efficacy and acceptable tolerability in AOM/DSS-induced colorectal cancer models [19, 25, 26]. The experiment was conducted for a period of 16 weeks, of which 6 weeks were for the induction of tumours, and the rest were the treatment phase only (Fig. 1a). Body weights and disease activity index (DAI) scores were recorded weekly throughout the experimental period. At the end of the experimental period, mice were euthanised under standard ketamine-xylazine anaesthesia (10 and 90 mg/kg, i.p.). Blood was collected after cardiac perfusion; serum was isolated and stored at -20℃ for estimation of the biomarkers of liver injury along with liver tissue. The colons were excised, cleared of any faecal matter, measured and tumours were counted microscopically, and finally processed for histology, immunohistochemistry, and gene expression analysis. The length of the colon was assessed right after excision by gently elongating it without undue tension prior to fixation; the photographs shown in Fig. 1d are representative images and were not used for length measurement.

### Disease Activity Indexing (DAI) and tumour numbers in the colon

The severity of CRC was monitored using the standard DAI scores according to the diagnostic criteria reported previously [27]. The animals were thoroughly observed for any of the following physiological changes, including loss of body weight, rates of defecation, stool consistency, occurrence of rectal bleeding, mobility and feeding behaviour, etc., and scored accordingly (Table 2 and [27]). Tumour burden was quantified by counting all the visible tumours in the excised colons under a microscope, and the average number of tumours per group was calculated [19].

**Table 2:** Disease Activity Index Score

Score	Weight Loss (%)	Stool Consistency	Gross Bleeding
0	None	Normal	Negative
1	1 to 5	-	-
2	6 to 10	Loose Stools	Hemoccult Positive
3	11 to 15	-	-
4	More than 15	Watery diarrhoea	Bleeding

### Histopathological study of the colon

For the histopathological analysis, the colons were first excised, then rinsed in 1X phosphate-buffered saline, and finally fixed in 10% neutral buffered formalin for 24 h. The formalin-fixed colon tissues were washed, dehydrated, and embedded in paraffin for the preparation of histological sections. Sections were then cut into 4 μm thickness and stained with routine Haematoxylin and Eosin (H&E) method to assess various histopathological alterations, including the infiltration of inflammatory cells, glandular adenoma, atypical dysplasia & presence of any adenocarcinoma lesions. Scoring for each sample was done ranging from 0 to 4 (0: no characters; 1: only 1 character present; 2,3, and 4 scores representing 2, 3 and 4 characters, respectively). A few sections of colon from each group were stained with Alcian blue/ Nuclear fast red (AB/NFR) for quantification of goblet cells per crypt [28]. All the histopathological scorings were calculated in a blinded manner by examining 5 slides per animal per group under high power fields (×40) of a microscope. All the histopathological analyses, wherever required, were performed using equipment from Leica Microsystems (Switzerland) Limited with the following application: Leica Application Suite, Version 4.2.0 [Build 607], License Number LAS14811. The objective lenses of the system were Leica N PLAN ×10 (NA 0.25 mm, achromatic optical correction, working distance approx. 17.7 mm), and the objective lens Leica N PLAN ×40 (NA 0.65 mm, achromatic objective class, N PLAN, working distance approx. 0.36 mm). The microsystem included a camera with following specifications: a Leica DFC295-500424310 digital microscope colour camera offering a standard resolution of 2048 × 1536 pixels. Version information: Twain Version 7.7.0.0, FxLib 5.1.0.10570, Firmware 1.0.11. Type of acquisition software included in the system was Leica Application Suite (LAS v4.x).

### Immunohistochemical analysis

For the immunohistochemical analysis of different markers in the colon tissues, the paraffin-embedded sections were deparaffinized, rehydrated through a series of alcohols, and antigens were retrieved at 90 °C for 45 min. The sections were then blocked with 3% BSA at room temperature to minimise nonspecific binding and incubated overnight with the primary antibody at 4 °C (dilution as per manufacturer’s validation, Table 1). The following day, those sections were incubated again with HRP-conjugated secondary antibodies for 30 min. Visualization was achieved using 3,3-diaminobenzidine (DAB), and the signals were enhanced with 1% CuSO₄ solution for 3 min. The slides were subsequently counterstained with haematoxylin, dehydrated through graded alcohol, cleared in xylene, and mounted with DPX for microscopic analysis. Localisation of PCNA (Proliferating cell nuclear antigen), Cleaved Caspase-3, CD31 (Cluster of differentiation 31) and VEGFR2 (Vascular endothelial growth factor receptor 2) was observed at different parts of the colon, and the differences were analysed among the groups by quantification of positive cells/area per sample after analysing at least 10 high-power microscopic fields (HPF) per animal. Scoring for each sample was done based on four categories of areas of staining: 0 referred to negative; 1- low (below 25%); 2- medium (between 25% and 50%); 3- high (above 50%). Published studies utilizing the antibodies have been cited in the manuscript to further substantiate their specificity [29–32]. Established manufacturer validation, together with the supporting literature confirms the reliability and specificity of the antibodies employed in this study.

### Gene expression analyses by the qRT-PCR method

For the expression analysis of different genes, the total RNA was extracted from colon tissues of different groups of animals using Trizol and reverse transcribed to mRNA with the PrimeScript™ RT reagent kit (TaKaRa, Tokyo, Japan, RR037A). qRT-PCR was performed through a Tata MD CHECK Express system using SYBR Green chemistry (TB Green Premix Ex Taq II, RR820A). GAPDH was used as the internal control for normalisation, and the relative expression levels were calculated using MyGo Pro PCR Software v.3.6. Primer sequences for the target mouse genes were sourced from previously published literature, verified on the NCBI Primer-BLAST for specificity and accuracy, and are presented in Table 3.

**Table 3:** List of different gene-specific primers for the mouse cDNA

Sl. No	Gene	Primer Sequence from 5’-3’	References
1.	*GAPDH*	F-CTCCCACTCTTCCACCTTCG R- GCCTCTCTTGCTCAGTGTCC	[33]
2.	*β-catenin*	F-GCTGACCTGATGGAGTTGGA R- GCTACTTGCTCTTGCGTGAA	[34]
3.	*Cyclin D1*	F-GCAGAAGGAGATTGTGCCATCC R- AGGAAGCGGTCCAGGTAGTTCA	[35]
4.	*E-cadherin*	F-TCCAACAGGGACAAAGAAACAA R- TGACACGGCATGAGAATAGAGG	[36]
5.	*CD31*	F-ACTTCTGAACTCCAACAGCGA R- CCATGTTCTGGGGGTCTTTAT	[37]
6.	*VEGFR2*	F-ACGAGGAGAGAGGGTCATCT R- GACACACTCTCCTGCTCAGT	[38]

### Estimation of serum biomarkers of liver injury and liver histopathology

Blood was collected in sterilised tubes, incubated at RT (30 min), and serum was separated after centrifugation at 3000 rpm for 10 min. The clear and non-haemolyzed sera were used to determine the concentration of ALT, AST, and ALP expressed in U/L and TBIL expressed in mg/dl. All biochemical estimation was conducted using standard kits based on previously reported methodology (ALT & AST: Modified IFCC method; ALP: Modified Bessey-Lowry method; TBIL: Modified Jendrassik & Grof’s method).

Liver tissues were also processed for histopathological analysis as previously described, stained for quantification of histopathological scores using the following characters such as infiltration of inflammatory cells in periportal areas, necrosis and sinusoidal dilatation. Scoring for each sample was done ranging from 0 to 3 (0: no changes, 1: presence of any one of the selected criteria, 2: presence of any two of the selected criteria and 3: presence of all the selected criteria). All the histopathological scorings were calculated in a blinded manner by examining 5 slides per animal per group in high-power fields.

### Statistical analyses

Data generated in the experiment were represented as mean ± SD, and the statistical differences of mean among the groups were compared using one-way ANOVA, followed by post hoc Tukey test, wherever applicable. The ordinal data were presented as median with interquartile range and statistical analysis was performed using the Kruskal–Wallis test followed by Dunn’s post hoc multiple comparisons test. *p* < 0.05 was considered statistically significant. All statistical analyses were conducted using GraphPad Prism statistical software V.10.0.

## Results

### Effect of combination therapy of EGCG with IRN on body weight, disease activity index, colon length and tumour numbers in AOM/DSS induced CRC model

At the end of the experiment period, significant weight loss was observed in the PC group compared to the NC group (*** *p* < 0.001; Fig. 1b). However, significant weight gain was observed after treatment with EGCG alone (** *p* < 0.01) or in combination with IRN compared to the PC group (*** *p* < 0.001; Fig. 1b). DAI score was found to be elevated in both PC and IRN groups of animals compared to the NC group (*** *p* < 0.001), however DAI score was prominently reduced in both EGCG and combined IRN+EGCG groups of animals (*** *p* < 0.001), (Fig. 1c). Figure 1d demonstrates representative images illustrating gross morphology of colon of different treatment groups. The colon length was significantly reduced in PC group compared to the NC group (*** *p* < 0.001), whereas all other groups exhibited significant treatment related increase in colon length when compared to the PC group (Fig. 1e). As expected, a significantly higher number of tumours per animal was recorded in the PC group (*** *p* < 0.001) compared to all other groups of animals (Fig. 1f). However significant treatment related decrease in the number of tumours was recorded in all other treatment groups and the least number of tumours being recorded in the combined IRN & EGCG treated group of animals (*** *p* < 0.001; Fig. 1f).

### Effect of EGCG supplementation alone or in combination with IRN treatment on colon histopathology

**Fig. 2:**
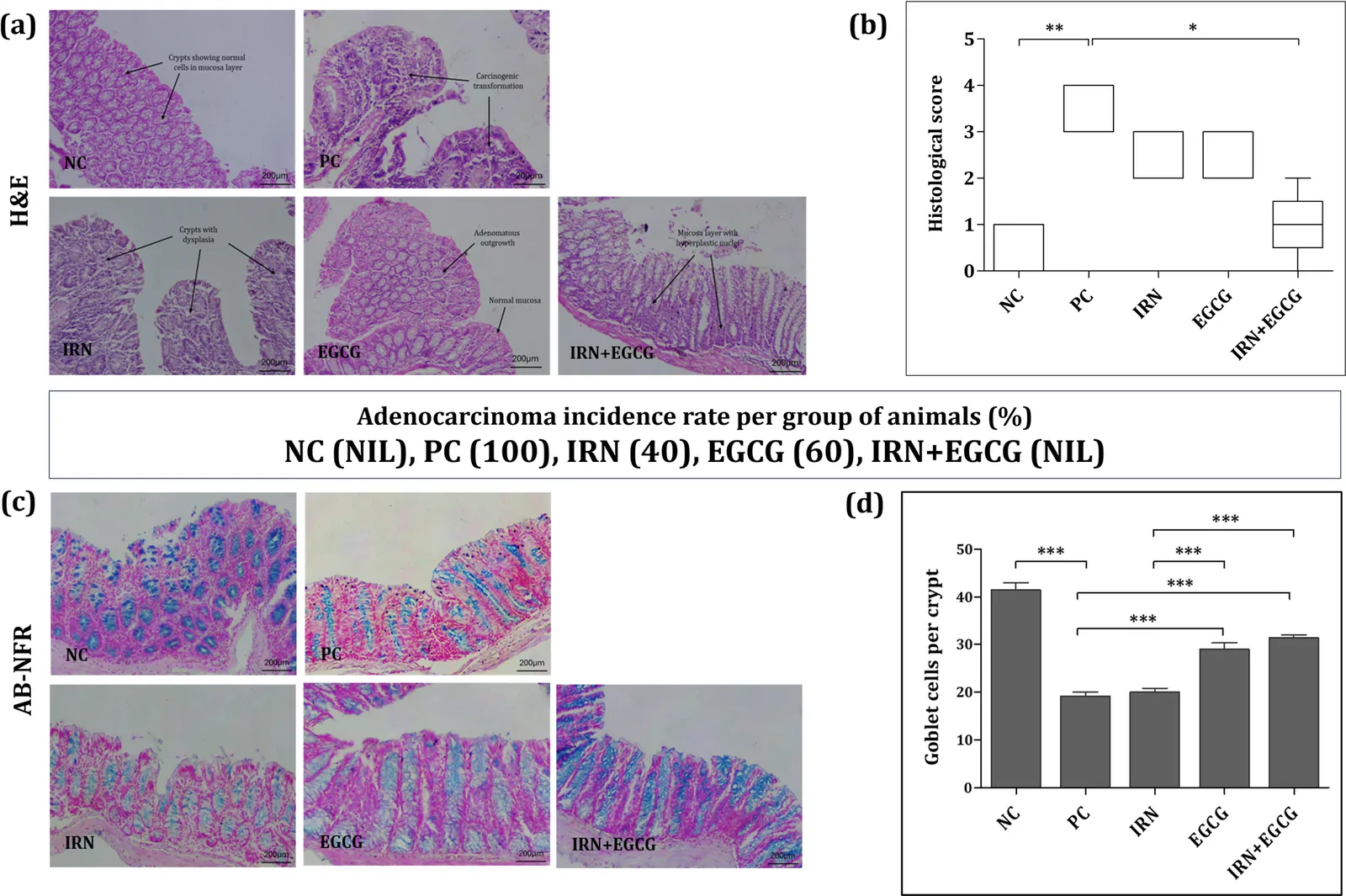
A comparative representation of (**a**) H&E-stained histological sections from different treated groups of animals (scale bar 200 μm), (**b**) histopathological scoring of the colon per high power field (HPF), (**c**) AB/NFR staining to demonstrate mucin containing goblet cells (scale bar 200 μm) and (**d**) Quantification of goblet cells per crypts in 5 groups of animals. *** *p* < 0.001, ** *p* < 0.01, * *p* < 0.05

Representative histopathological images of the colon and corresponding histopathological scores for each group of animals were presented in Fig. 2a and b. The colon sections from NC group exhibit normal histoarchitecture with intact mucosal strata, well defined regular crypts, organised muscular layer and surface epithelium (Fig. 2a). Meanwhile, the colons from PC group were found to have noticeable carcinogenic transformations involving hyperplasia, high grade dysplasia, adenoma and adenocarcinoma and showed elevated histological score compared to NC (** *p* < 0.01, Fig. 2a & b), which was significantly reduced in both IRN and EGCG treated group of animals (Fig. 2a & b). However, the colon sections from combined IRN+EGCG treated group of animals, was found to exhibit only hyperplastic nuclei and a near normal histoarchitecture and a decreased histological score compared to PC (* *p* < 0.05) and other single treatment groups (Fig. 2a & b). Moreover, the percentage incidence of adenocarcinoma in different groups of animals was found to be NC (nil), PC (100), IRN (40), EGCG (60) & IRN+EGCG (nil).

Representative AB/NFR-stained sections of colon for quantification of goblet cells per crypt for each group of animals were presented in Fig. 2c and d. A significant reduction in the number of goblet cells in the colon tissues was recorded from both PC and IRN groups of animals compared to the NC group (*** *p* < 0.001; Fig. 2c & d). However, supplementation with EGCG alone or in combination significantly restored the number of goblet cells in both the EGCG and IRN+EGCG treated group of animals (*** *p* < 0.001; Fig. 2c & d).

### Effect of EGCG supplementation alone or in combination with IRN treatment on immunohistochemical expression of the PCNA and Cleaved caspase-3 and expression of key oncogenic markers (β-catenin, Cyclin D1 & E-cadherin)

**Fig. 3:**
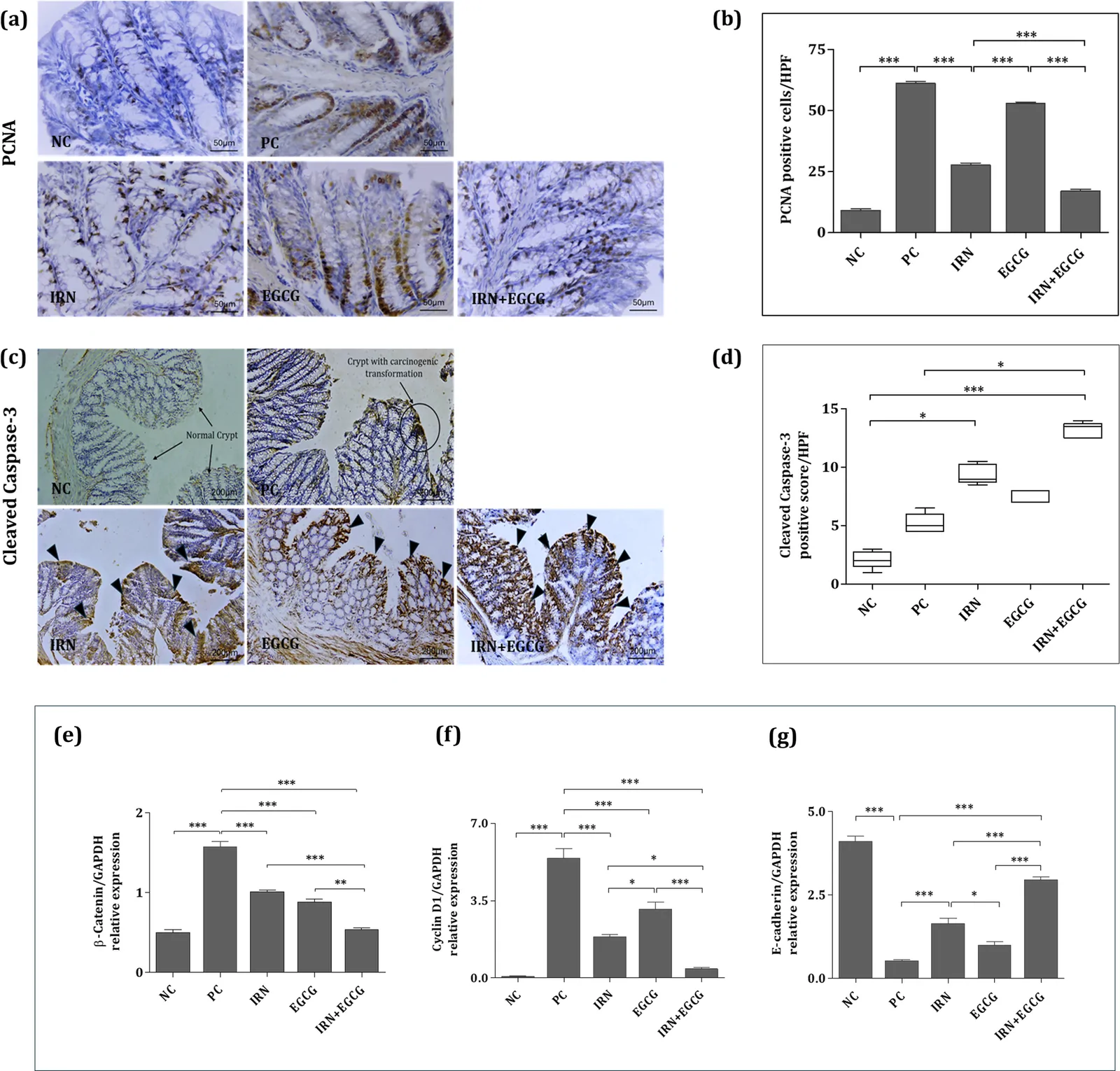
A comparative representation of immunohistochemical stained colon sections with (**a**) PCNA from different treated groups of animals (scale bar 50 μm), **b**) Quantification of PCNA positive cells per high power field (HPF), **c**) Cleaved Caspase-3-stained sections from different groups of animals (scale bar 200 μm) & **d**) Quantification of Cleaved caspase-3 positive areas per high power field (HPF). The relative mRNA expression of (**e**) β-catenin, (**f**) Cyclin D1 and (**g**) E-cadherin with reference to GAPDH. *** *p* < 0.001, ** *p* < 0.01, * *p* < 0.05

Immunohistochemical analysis of PCNA, the marker for cellular proliferation, recorded a significantly higher number of PCNA positive cells in the colon tissues of PC group compared to the NC group (*** *p* < 0.001), however the number of PCNA positive cells was found to be significantly reduced in all treatment groups compared to PC group. Interestingly, only EGCG treatment was found to have less impact on PCNA occurrence compared to IRN treated group (*** *p* < 0.001). However, compared to IRN only group, combined IRN & EGCG group was found to have more significant effect on the reduction of PCNA positive cells in the colon (*** *p* < 0.001; Fig. 3a & b). Antibodies against cleaved caspase-3, an apoptotic marker, were used to compare the occurrence of cell death in colon tissues among different treatment groups. The combined IRN & EGCG treated group was found to have significantly highest colon area with positive signals for cleaved caspase-3, suggesting the prominent occurrence of apoptosis within this group of animals compared to only EGCG and IRN monotherapy groups and NC (*** *p* < 0.001, Fig. 3c & d).

Relative mRNA expression of key oncogenic markers was presented in Fig. 3e-g. The qRT-PCR analysis of oncogenic biomarkers recorded upregulated expression of β-catenin and Cyclin D1 in the PC group when compared to the NC (*** *p* < 0.001; Fig. 3e & f). In the combined IRN & EGCG treated group, levels of both β-catenin and Cyclin D1 were significantly reduced when compared to PC (*** *p* < 0.001) and all other treatment groups (Fig. 3e & f). However, a significant decline in relative expression of E-cadherin was observed in the PC group compared to NC group (*** *p* < 0.001), but its expression was restored in the combined IRN & EGCG treated group when compared to other single treatment groups (*** *p* < 0.001; Fig. 3g).

### Effect of EGCG supplementation alone or in combination with IRN treatment on expression of the key angiogenesis marker, VEGFR2 and CD31

**Fig. 4:**
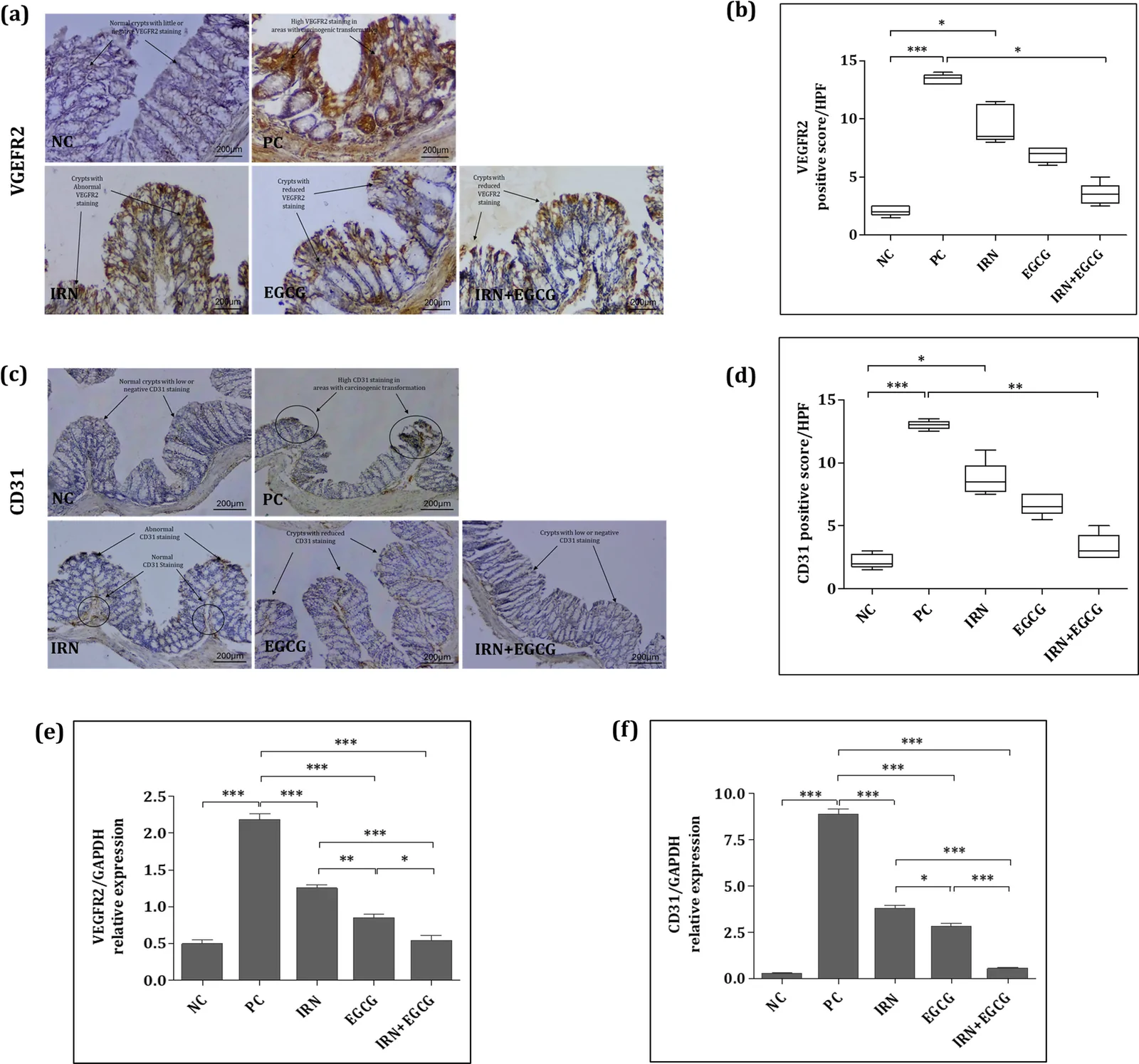
A comparative representation of immunohistochemical detection and scoring of (**a**) & (**b**) VEGFR2 and (**c**) & (**d**) CD31 in colon sections from different groups of animals (scale bar 200 μm). The relative mRNA expression of (**e**) VEGFR2 and (**f**) CD31 in colon tissues of different groups relative to GAPDH. *** *p* < 0.001, ** *p* < 0.01, * *p* < 0.05

Immunohistochemical quantification of CD31 and VEGFR2, the markers for angiogenesis recorded significant increase in positive staining in the colon tissues of PC group (***p < 0.001) compared to NC, indicating tumour angiogenesis, whereas it was rarely detected in the colonic tissue of NC group. Notably, a significant reduction in VEGFR2 (4a-b) as well as CD31 (4c-d) expressions was observed in all treated groups, however the combined IRN & EGCG treated groups recorded highest significant reduction in the expression of both VEGFR2 and CD31 (Fig. 4a-b, *p < 0.05 & 4c-d, **p < 0.01).

In terms of gene expression analysis, a significant upregulation of both VEGFR2 and CD31 was recorded for PC group over NC group (*** *p* < 0.001; Fig. 4e & f). A significant treatment related reduction in the expression of both VEGFR2 and CD31 was recorded across all the treatment groups, but the combined treatment of IRN & EGCG was found to more efficient in suppression of gene expression of both VEGFR2 and CD31 (*** *p* < 0.001; Fig. 4e and f).

### Effect of EGCG supplementation alone or in combination with IRN treatment on expression of serum biomarker of liver injury and liver histopathology

**Fig. 5:**
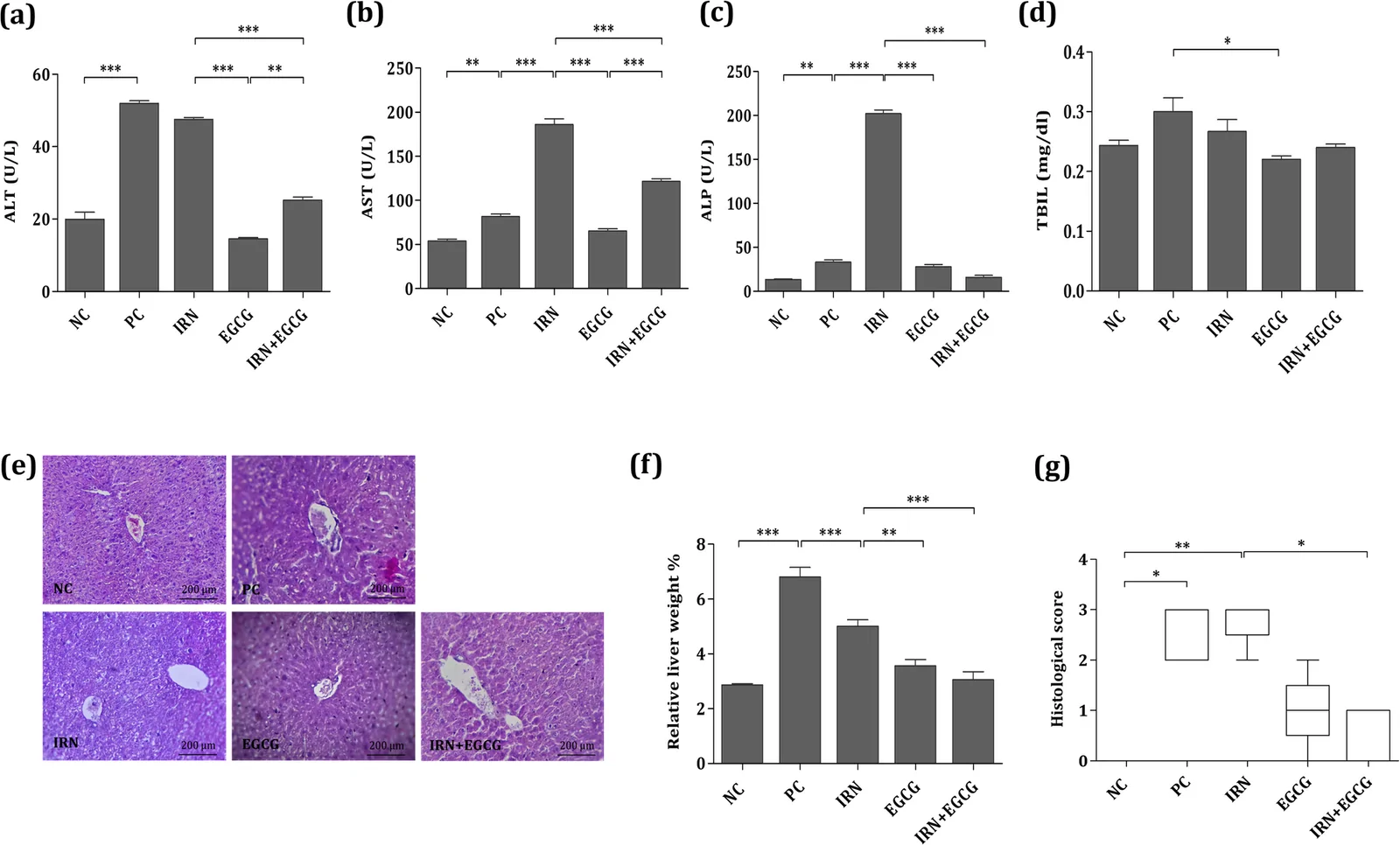
Estimation of serum biomarkers of liver injury (**a**) ALT (Alanine Aminotransferase) (U/L), (**b**) AST (Aspartate Aminotransferase) (U/L), (**c**) ALP (Alkaline Phosphatase) (U/L) & (**d**) TBIL (Total Bilirubin) (mg/dl) in different group of animals. A comparative representation of (**e**) H&E-stained histological liver sections from different groups of animals (scale bar 200 μm), (**f**) relative liver weight (%) and (g) histological scoring for evaluation of treatment-associated liver injury under per high-power field (HPF). *** *p* < 0.001, ** *p* < 0.01, * *p* < 0.05

The serum biomarkers of liver were analysed in all treatment groups of animals and presented in Fig. 5a and d. Treatment with IRN caused a significant increase in the serum level of ALT, AST and ALP when compared to all other groups of animals except the PC group (Fig. 5a-c). However, treatment with EGCG alone or in combination with IRN significantly reduced the serum level of ALT, AST and ALP compared to the IRN treated group (*** *p* < 0.001; Fig. 5a and c). No significant changes in serum level of TBIL was recorded except between PC and EGCG treated group of animals (* *p* < 0.05; Fig. 5d).

Representative H&E-stained histological sections of liver tissue from different treatment groups with relative liver weight and histopathological scores are presented in Fig. 5e and g. Relative liver weight was found to significantly elevated in both PC and IRN treated animal groups (***p < 0.001), whereas compared to IRN group, supplementation of EGCG alone or with IRN significantly reduced the relative liver weight in the experimental animal groups (** *p* < 0.01, *** *p* < 0.001; Fig. 5f). IRN treatment caused hepatocyte necrosis and peri- and centrilobular accumulation of inflammatory cells and dilatation of sinusoidal space which was reversed in groups supplemented with EGCG (* *p* < 0.05; Fig. 5e and g).

## Discussion

CRC poses a global health burden with chemotherapeutic treatment remaining as the cornerstone for CRC management. The major chemotherapeutic drugs used for management and treatment of CRC include cytotoxic drugs like 5-fluorouracil, irinotecan, oxaliplatin, floxuridine, etc., and other angiogenic inhibitors such as Avastin, Erbitux, etc., for more advanced form of CRC [39]. However, with chemotherapeutic treatment, it comes with side-effects including fatigue, nausea, vomiting, mouth sores, hair loss, diarrhoea, and weakened immunity. As such focus is on to develop bioactive compounds from natural sources which might lower side effects of chemotherapeutic drugs. Many bioactive molecules such as curcumin, resveratrol, EGCG, quercetin and baicalein etc., have been reported to possess strong anti-cancer activity, protecting the normal cells from chemotherapy related toxicities [40]. EGCG is a well-studied polyphenol derived from green tea and act as an antioxidant, anti-angiogenic, anti-tumour agent and reported to induce anticancer activity via induction of apoptosis by activating caspases and cell cycle inhibition [41]. EGCG was also reported to cause apoptotic death of various cancer types either alone or in combination with other chemotherapeutic drugs and it enhanced the chemotherapeutic efficacy in recurrent and recalcitrant malignancy [42].

As the number of cases continue to rise globally, search for alternate strategies such as combination therapies with natural bioactive compounds are increasingly being investigated for enhancing chemotherapeutic efficacy and treating various disorders [43–46]. The combination therapy is advantageous over monotherapy as it targets key pathways either in synergistic or additive manner to reduce tumour burden [47].

In the case of CRC, combination therapy of chemotherapeutic drugs with natural compounds and bio-actives has proven to be effective by enhancing anti-tumour efficacy, mitigating the development of drug resistance, and promoting more substantial tumour regression compared with monotherapy [25, 48]. Previous reports confirmed the efficacy of combining chemotherapy with natural bioactive molecules in the management of different cancer types. The efficacy of combination of resveratrol with paclitaxel against breast cancer cells in xenograft model and quercetin with paclitaxel against ovarian cancer cells has already been reported previously [49, 50].

Similarly, in the present study it was evident that the combination of IRN with EGCG was more potent than either IRN or EGCG in reducing tumour burden in the AOM/DSS model of CRC in mice. Interestingly, contrary to many previous in vitro findings, only EGCG treatment was found not to be sufficient and less potent than IRN in reducing tumour burden in AOM/DSS model of CRC [51]. This might be due to low bioavailability, stability and rapid elimination of EGCG from in vivo system [52].

In CRC, the interplay among β-catenin, E-cadherin, and cyclin D1 has been reported to play an important role in tumourigenesis. Elevated β-catenin levels functions as a transcriptional regulator, promoting overexpression of the cell cycle protein cyclin D1. Concomitantly, loss or dysfunction of membrane-bound E-cadherin during carcinogenesis facilitates the release and accumulation of β-catenin, thereby promoting proliferation while diminishing cell-cell adhesion [53]. In the present study, combined treatment with IRN & EGCG significantly improved disease outcomes compared not only to positive control but also over the monotherapy groups by significantly reducing tumour burden, improving histoarchitecture and by restoration of goblet cell numbers in colonic tissues. This was also confirmed from immunohistochemical quantification of PCNA positive cells and reduced gene expression of cyclin D1 and β-catenin and restored levels of E-cadherin in the colon tissues of combined treated group. The present findings are in alignment with previously published reports [19, 54].

Apoptosis is a key mechanism by which most chemotherapeutic drugs act to reduce tumour burden. IRN, is a class I topoisomerase inhibitor which mediates chemotherapeutic effect by inducing single strand DNA breaks and finally triggers apoptosis by activating caspase-3 [55]. EGCG is also reported to induce cancer cell apoptosis by downregulating anti-apoptotic proteins and by upregulating pro-apoptotic proteins [56]. In the present study, immunohistochemical quantification revealed the expression of cleaved caspase-3 was found to be elevated in all treated groups; however, the expression was significantly higher in combined IRN & EGCG treated group compared to only IRN or EGCG treated group. This provides another direct evidence of additive effect of IRN and EGCG in reducing tumour burden in AOM/DSS model of CRC.

Angiogenesis which involves the formation of blood vessels is a complex and tightly regulated process which when disturbed can enhance tumour growth, invasion and metastasis. Since tumour angiogenesis has an important role to play in carcinogenesis, therefore, many such compounds exhibiting anti-angiogenic properties have been investigated so far to play a therapeutic role in various types of cancers including CRC. Considerable attention has shifted towards natural compounds and bio-actives possessing anti-angiogenic effect on various cancer types [48, 57]. Notably, it has been reported earlier that among the four major green tea catechins, EGCG is the most potent inhibitor of angiogenesis and combination treatment with curcumin and other polyphenols has resulted in enhanced anti-angiogenic effect in various cancer models [58].

In the current study, the combined therapy of IRN+EGCG had a marked effect on the reduction of key angiogenic markers, VEGFR2 and CD31, compared to single treatment groups and was illustrated using RT-qPCR and immunohistochemical techniques. These results suggested that EGCG could inhibit tumour angiogenesis in vivo in the presence of IRN more robustly and might also lead to regression of existing tumour vessels. According to many previous in vitro studies on a variety of cancers, including hepatocellular, breast, and prostate, EGCG has been reported to be a potent anti-angiogenic agent that has potential to block angiogenesis at quantities attainable by tea drinking [59–61].

The effectiveness of chemotherapy is often restricted by major limitations such as IRN-treatment induced hepatotoxicity that is also reflected by elevated serum transaminases as reported previously [19, 62]. Like that, in the present study IRN treated group of animals displayed treatment related adverse hepatotoxicity as reflected from elevated serum level of ALT, AST and ALP and histopathological alteration of liver tissues. IRN and its active metabolite SN-38 is reported to produce reactive oxygen species and mitochondrial dysfunction thereby leading to accumulation of fats in the hepatocyte [63]. However, in groups of animals treated with EGCG alone or in combination with IRN, significantly reduced serum level of ALT, AST and ALP was observed in the current study which was further reflected from improved histoarchitecture of liver tissues in these groups of animals. The present study did not find any significant treatment related effects on total serum bilirubin level (TBIL). While irinotecan may induce hepatic injury, previous studies have also reported that temporary, mild-to-moderate elevations in bilirubin were observed in a limited number of patients and these instances were frequently not associated with severe jaundice [64]. These findings hence support the previously reported hepatoprotective nature of green tea polyphenols in IRN induced toxicity [19]. In previous findings, the hepatoprotective activity of EGCG was attributed to its ability to scavenge free radical due to its antioxidant property and downregulation of pro-inflammatory markers [62, 65]. Similar studies depicting the hepatoprotective effects of natural plant compounds have also been reported earlier in vivo [66]. To summarize, findings of the present study demonstrated that supplementation of EGCG with IRN has a potential in suppression of key tumour-associated angiogenic factors and reduced tumour growth, improved overall symptoms and treatment-associated liver injury in the AOM/DSS induced CRC mice model.

## Conclusion

Conventional chemotherapeutic treatment causes some severe side effects, mainly due to drug or radiation damage to the cells of the intestinal tract, resulting in inflammation of the mucosa. In the present study, EGCG in combination with IRN inhibited tumourigenesis, enhanced its therapeutic efficacy and demonstrated strong inhibition of tumour-associated angiogenesis markers in AOM/DSS model of CRC. In summary, the combination treatment demonstrated enhanced anti-tumour activity and stronger inhibition of key tumour-associated angiogenesis markers compared to either single treatment administration.

### Limitations of the present study

Mechanistic validation using CRC cell lines and quantitative protein analyses (e.g., Western blotting) could not be performed since this study is focused on molecular and histoarchitectural validation using qRT-PCR and is limited to in vivo studies, however, incorporation of cell line specific studies and validation at protein level in future will further substantiate the present findings. Moreover, extended survival analysis beyond the established 16-week threshold was not feasible as all subjects were humanely sacrificed in accordance with the sanctioned ethical guidelines, however subsequent studies with extended survival outcomes may further validate the longevity of the combined treatment benefits. Furthermore, the potential toxicity of irinotecan and EGCG in healthy mice has not been assessed and remains to be investigated in future studies. Additionally, further validation using a tumour angiogenesis model are needed to investigate the potential mechanisms involved in the combination chemotherapeutic effect between IRN and EGCG to strengthen the hypothesis.

## Supplementary Information

Supplementary Material 1.

## Data Availability

All data supporting the findings of this study are available within the paper.

## Abbreviations

AOM: Azoxymethane
ALT: Alanine transaminase
AST: Aspartate aminotransferase
ALP: Alkaline phosphatase
CRC: Colorectal cancer
CD31: Cluster of differentiation 31/ Platelet endothelial cell adhesion molecule
DAI: Disease activity index
DSS: Dextran sulphate sodium
EGCG: Epigallocatechin-3-gallate
HPF: High Power microscopic Field
IHC: Immunohistochemistry
IRN: Irinotecan
IRN+EGCG: Irinotecan + Epigallocatechin-3-gallate
NC: Normal control
PC: Positive Control
PCNA: Proliferating Cell Nuclear Antigen
TBIL: Total bilirubin
VEGF: Vascular endothelial growth factor
VEGFR2: Vascular endothelial growth factor receptor 2
