# Supplementary material for: Epigallocatechin-3-Gallate, the green tea polyphenol suppresses expression of CD31 and VEGFR2 and enhances the chemotherapeutic potential of irinotecan in AOM/DSS model of colorectal cancer in mice
Source: J Egypt Natl Canc Inst. 2026 Sep 17;38:73. doi: 10.1186/s43046-026-00412-4 (PMC13586132; doi:10.1186/s43046-026-00412-4)
Supplement: Supplementary file 1 [file 43046_2026_412_MOESM1_ESM.docx]

**SUPPLEMENTARY TABLE 1: TUKEY’S MULTIPLE COMPARISON TEST OF ANIMAL BODY WEIGHT**

| WEEKS | GROUPS | Significant? P < 0.05? | Summary |
| --- | --- | --- | --- |
| WEEK 0 | NC vs PC; NC vs IRN; NC vs EGCG; NC vs IRN+EGCG; PC vs IRN; PC vs EGCG; PC vs IRN+EGCG; IRN vs EGCG; IRN vs IRN+EGCG; EGCG vs IRN+EGCG | No; No; No; No; No; No; No; No; No; No | ns; ns; ns; ns; ns; ns; ns; ns; ns; ns |
| WEEK 1 | NC vs PC; NC vs IRN; NC vs EGCG; NC vs IRN+EGCG; PC vs IRN; PC vs EGCG; PC vs IRN+EGCG; IRN vs EGCG; IRN vs IRN+EGCG; EGCG vs IRN+EGCG | No; No; No; No; No; No; No; No; No; No | ns; ns; ns; ns; ns; ns; ns; ns; ns; ns |
| WEEK 2 | NC vs PC; NC vs IRN; NC vs EGCG; NC vs IRN+EGCG; PC vs IRN; PC vs EGCG; PC vs IRN+EGCG; IRN vs EGCG; IRN vs IRN+EGCG; EGCG vs IRN+EGCG | No; No; No; No; No; No; No; No; No; No | ns; ns; ns; ns; ns; ns; ns; ns; ns; ns |
| WEEK 3 | NC vs PC; NC vs IRN; NC vs EGCG; NC vs IRN+EGCG; PC vs IRN; PC vs EGCG; PC vs IRN+EGCG; IRN vs EGCG; IRN vs IRN+EGCG; EGCG vs IRN+EGCG | No; No; No; No; No; Yes; Yes; Yes; Yes; No | ns; ns; ns; ns; ns; *; **; *; **; ns |
| WEEK 4 | NC vs PC; NC vs IRN; NC vs EGCG; NC vs IRN+EGCG; PC vs IRN; PC vs EGCG; PC vs IRN+EGCG; IRN vs EGCG; IRN vs IRN+EGCG; EGCG vs IRN+EGCG | No; No; No; No; No; No; Yes; No; Yes; No | ns; ns; ns; ns; ns; ns; **; ns; *; ns |
| WEEK 5 | NC vs PC; NC vs IRN; NC vs EGCG; NC vs IRN+EGCG; PC vs IRN; PC vs EGCG; PC vs IRN+EGCG; IRN vs EGCG; IRN vs IRN+EGCG; EGCG vs IRN+EGCG | No; No; No; No; No; No; No; No; No; No | ns; ns; ns; ns; ns; ns; ns; ns; ns; ns |
| WEEK 6 | NC vs PC; NC vs IRN; NC vs EGCG; NC vs IRN+EGCG; PC vs IRN; PC vs EGCG; PC vs IRN+EGCG; IRN vs EGCG; IRN vs IRN+EGCG; EGCG vs IRN+EGCG | No; No; No; No; No; No; No; No; No; No | ns; ns; ns; ns; ns; ns; ns; ns; ns; ns |
| WEEK 7 | NC vs PC; NC vs IRN; NC vs EGCG; NC vs IRN+EGCG; PC vs IRN; PC vs EGCG; PC vs IRN+EGCG; IRN vs EGCG; IRN vs IRN+EGCG; EGCG vs IRN+EGCG | Yes; No; No; No; No; No; No; No; No; No | *; ns; ns; ns; ns; ns; ns; ns; ns; ns |
| WEEK 8 | NC vs PC; NC vs IRN; NC vs EGCG; NC vs IRN+EGCG; PC vs IRN; PC vs EGCG; PC vs IRN+EGCG; IRN vs EGCG; IRN vs IRN+EGCG; EGCG vs IRN+EGCG | Yes; No; No; No; Yes; No; Yes; No; No; No | ***; ns; ns; ns; *; ns; *; ns; ns; ns |
| WEEK 9 | NC vs PC; NC vs IRN; NC vs EGCG; NC vs IRN+EGCG; PC vs IRN; PC vs EGCG; PC vs IRN+EGCG; IRN vs EGCG; IRN vs IRN+EGCG; EGCG vs IRN+EGCG | Yes; No; No; No; Yes; Yes; Yes; No; No; No | ***; ns; ns; ns; **; *; ***; ns; ns; ns |
| WEEK 10 | NC vs PC; NC vs IRN; NC vs EGCG; NC vs IRN+EGCG; PC vs IRN; PC vs EGCG; PC vs IRN+EGCG; IRN vs EGCG; IRN vs IRN+EGCG; EGCG vs IRN+EGCG | Yes; No; No; No; Yes; Yes; Yes; No; No; No | ***; ns; ns; ns; **; **; ***; ns; ns; ns |
| WEEK 11 | NC vs PC; NC vs IRN; NC vs EGCG; NC vs IRN+EGCG; PC vs IRN; PC vs EGCG; PC vs IRN+EGCG; IRN vs EGCG; IRN vs IRN+EGCG; EGCG vs IRN+EGCG | Yes; No; No; No; No; Yes; Yes; No; No; No | ***; ns; ns; ns; ns; *; ***; ns; ns; ns |
| WEEK 12 | NC vs PC; NC vs IRN; NC vs EGCG; NC vs IRN+EGCG; PC vs IRN; PC vs EGCG; PC vs IRN+EGCG; IRN vs EGCG; IRN vs IRN+EGCG; EGCG vs IRN+EGCG | Yes; Yes; No; No; No; Yes; Yes; No; Yes; No | ***; **; ns; ns; ns; **; ***; ns; *; ns |
| WEEK 13 | NC vs PC; NC vs IRN; NC vs EGCG; NC vs IRN+EGCG; PC vs IRN; PC vs EGCG; PC vs IRN+EGCG; IRN vs EGCG; IRN vs IRN+EGCG; EGCG vs IRN+EGCG | Yes; Yes; No; No; Yes; Yes; Yes; No; Yes; No | ***; *; ns; ns; ***; ***; ***; ns; **; ns |
| WEEK 14 | NC vs PC; NC vs IRN; NC vs EGCG; NC vs IRN+EGCG; PC vs IRN; PC vs EGCG; PC vs IRN+EGCG; IRN vs EGCG; IRN vs IRN+EGCG; EGCG vs IRN+EGCG | Yes; Yes; No; No; Yes; Yes; Yes; No; Yes; No | ***; *; ns; ns; ***; ***; ***; ns; *; ns |
| WEEK 15 | NC vs PC; NC vs IRN; NC vs EGCG; NC vs IRN+EGCG; PC vs IRN; PC vs EGCG; PC vs IRN+EGCG; IRN vs EGCG; IRN vs IRN+EGCG; EGCG vs IRN+EGCG | Yes; Yes; Yes; No; Yes; Yes; Yes; No; Yes; No | ***; ***; *; ns; ***; ***; ***; ns; **; ns |
| WEEK 16 | NC vs PC; NC vs IRN; NC vs EGCG; NC vs IRN+EGCG; PC vs IRN; PC vs EGCG; PC vs IRN+EGCG; IRN vs EGCG; IRN vs IRN+EGCG; EGCG vs IRN+EGCG | Yes; Yes; Yes; No; Yes; Yes; Yes; No; Yes; Yes | ***; ***; **; ns; ***; ***; ***; ns; **; * |
